# Regulation of Mitochondrial Respiration by VDAC Is Enhanced by Membrane-Bound Inhibitors with Disordered Polyanionic C-Terminal Domains

**DOI:** 10.3390/ijms22147358

**Published:** 2021-07-08

**Authors:** Tatiana K. Rostovtseva, Sergey M. Bezrukov, David P. Hoogerheide

**Affiliations:** 1 Program in Physical Biology, *Eunice Kennedy Shriver* National Institute of Child Health and Human Development, National Institutes of Health, Bethesda, MD 20892, USA; bezrukos@mail.nih.gov; 2 Center for Neutron Research, National Institute of Standards and Technology, Gaithersburg, MD 20899, USA; david.hoogerheide@nist.gov

**Keywords:** voltage-dependent anion channel, tubulin, α-synuclein, voltage gating, ATP transport, mitochondrial membranes, peripheral proteins, beta-barrel channels

## Abstract

The voltage-dependent anion channel (VDAC) is the primary regulating pathway of water-soluble metabolites and ions across the mitochondrial outer membrane. When reconstituted into lipid membranes, VDAC responds to sufficiently large transmembrane potentials by transitioning to gated states in which ATP/ADP flux is reduced and calcium flux is increased. Two otherwise unrelated cytosolic proteins, tubulin, and α-synuclein (αSyn), dock with VDAC by a novel mechanism in which the transmembrane potential draws their disordered, polyanionic C-terminal domains into and through the VDAC channel, thus physically blocking the pore. For both tubulin and αSyn, the blocked state is observed at much lower transmembrane potentials than VDAC gated states, such that in the presence of these cytosolic docking proteins, VDAC’s sensitivity to transmembrane potential is dramatically increased. Remarkably, the features of the VDAC gated states relevant for bioenergetics—reduced metabolite flux and increased calcium flux—are preserved in the blocked state induced by either docking protein. The ability of tubulin and αSyn to modulate mitochondrial potential and ATP production in vivo is now supported by many studies. The common physical origin of the interactions of both tubulin and αSyn with VDAC leads to a general model of a VDAC inhibitor, facilitates predictions of the effect of post-translational modifications of known inhibitors, and points the way toward the development of novel therapeutics targeting VDAC.

## 1. Introduction

The crucial role of mitochondrial outer membrane (MOM) permeability in maintaining an efficient metabolite exchange between mitochondria and cytoplasm in normal respiration is well-established, with VDAC recognized as the MOM key metabolite pathway and regulator. VDAC is the most abundant and perhaps the most studied protein of the MOM since its discovery in 1976 [1,2]. It is believed that the uniqueness of this relatively simple monomeric β-barrel channel mainly arises from its key position at the interface between a mitochondrion and the cytosol [3,4,5]. VDAC is not just a major conduit for the fluxes of most water-soluble metabolites and ions that must cross MOM to enter mitochondria and fuel oxidative phosphorylation, it also controls these fluxes [6,7,8]. Simultaneous access to strictly compartmentalized mitochondrial and cytosolic proteins endows VDAC with a unique regulating role over the vital communications between mitochondria and the cytosol. Therefore, it is perhaps unsurprising that VDAC has been implicated in a wide range of mitochondria-associated pathologies such as diabetes, hypertension, cardiovascular diseases, various cancers, and neurodegenerative disorders, such as Parkinson’s, Alzheimer’s, and amyotrophic lateral sclerosis [9,10,11,12,13,14].

The conceptual breakthrough demonstrating that VDAC is not just a molecular sieve and passive pathway for ions and nucleotides across MOM, but also an active controller of their transport, was made by Colombini’s group two decades ago by demonstrating that VDAC permeability to ATP and calcium can be regulated by voltage gating, the eponymous property of this channel [15,16]. In 1996 they found that VDAC’s voltage-induced “gated” states, which are more cation-selective and lower conducting for monovalent ions, are essentially impermeable to ATP, while the “open” state, which is anion-selective and conducts monovalent ions more readily, is permeable to ATP and sufficient to maintain all ATP efflux from mitochondria [16,17,18]. In 2007 Tan and Colombini showed that VDAC gated states have higher permeability for calcium and could conduct ~4 to 10-fold higher calcium flux than the open state [15]. The results of both studies suggest that VDAC’s open state facilitates flux of anionic metabolites but maintains a low calcium flux, thus promoting efficient oxidative phosphorylation. VDAC in its gated state, by contrast, drastically reduces metabolite transport but promotes calcium flux that impairs respiration, promotes the opening of the permeability transition pore, and endorses apoptotic signals. Thus, by voltage-induced gating, VDAC could regulate MOM permeability for metabolites and calcium.

A major reason for uncertainty regarding the physiological role of the VDAC gating mechanism for regulation of MOM permeability, however, is the source, magnitude, and regulation of the outer membrane potential in vivo. This issue has been addressed in multiple papers (see discussions in [3,19,20,21]). In a series of theoretical studies, Lemeshko estimated that the MOM potential could reach substantial values up to 50 mV, negative from the cytosolic side of MOM, enough to induce VDAC gating [22,23,24]. This model required tight binding of VDAC to hexokinase and a significant portion of VDAC to be either blocked by other protein(s) or a ligand or be in gated states [21,24]. It is thus reasonable to expect that if VDAC plays a crucial role in MOM permeability, there should exist mitochondrial or cytosolic endogenous VDAC regulators. Currently emerging proteomic, biochemical, and computational data identify VDAC as an interaction partner with a panoply of natural and synthetic compounds, anticancer and neuroprotective small molecule drugs, most of which are hydrophobic [25,26,27,28,29]. VDAC is also known to interact with a plethora of cytosolic proteins such as hexokinases, glycolytic enzymes, neuronal and cytoskeletal proteins [30,31,32,33,34], as well as with its neighbors in the MOM membrane, such as cholesterol transporter, TSPO [35,36], and Bcl-2-family proteins [37,38,39]. The most surprising observation highlighted by Reina and De Pinto in their comprehensive review [26] is the high diversity of these natural and synthetic ligands: there is no apparent structural, functional, or sequence similarity among them. The lack of high specificity to VDAC of most of the reported compounds and the absence of known molecular mechanisms of action create significant obstacles to using VDAC as a pharmacological target and developing a strategy for VDAC pharmacology. Considering VDAC’s unique role in mitochondrial physiology, this is a very unfortunate situation (see discussion in [40]).

**Figure 1:**
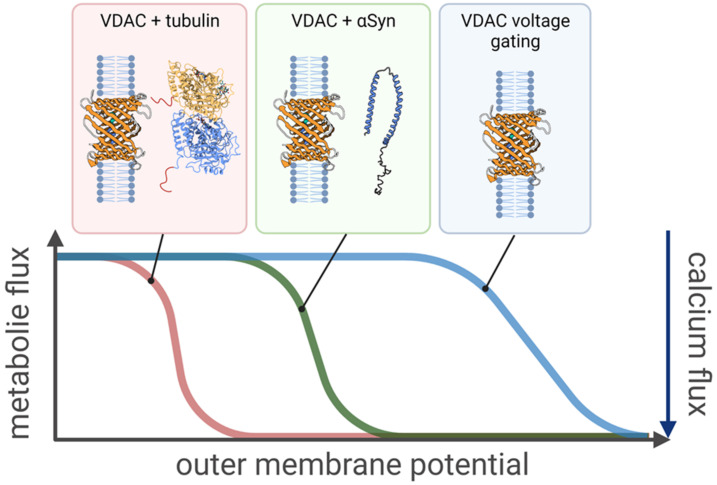
Increased sensitivity of VDAC (PDB ID: 3EMN) regulation to transmembrane potential in the presence of cytosolic regulators. Only a fraction of the outer membrane potential, relative to that necessary to induce channel voltage gating, is required for the effective control of VDAC-induced transport by tubulin and αSyn at concentrations as small as 10 nanomolar. The transport properties of the channel in response to the outer membrane potential, i.e., decrease in metabolite flux and the concomitant increase in calcium flux, remain qualitatively the same in all three cases. The “effective gating charge,” expressed as the steepness of the voltage dependence curve, is lowest for voltage gating (blue curve) and the highest for tubulin (red curve), with αSyn (green curve) in between.

In this review, we address this problem by discussing the molecular mechanism of VDAC regulation by two potent, but structurally and functionally distinct, cytosolic proteins: dimeric tubulin and α-synuclein (αSyn). Despite their structural dissimilarity, these two cytosolic proteins share a common feature: they both have polyanionic, disordered C-terminal tails (CTTs). At bulk concentrations as small as several nanomolar they regulate VDAC and, therefore, the MOM permeability with high efficiency. We show that these two physiologically unrelated proteins block the VDAC nanopore in vitro by the same physical mechanism: the capture of the CTTs into the pore by a transmembrane potential. When blocked by the CTTs, VDAC has similar metabolite and calcium transport properties as in its voltage-gated state. Thus, the net effect of the cytosolic regulators is to increase the sensitivity of VDAC closure to transmembrane potential (Figure 1). We show that the motion of each molecule in the nanopore can be modeled using a common physical framework, thus providing a unified description of VDAC regulation and a model of a general VDAC inhibitor or “docking protein”. Crucially, the driving force for the docking remains the transmembrane potential; surprisingly, biochemical compatibility plays a lesser role. We focus on two fundamental questions: what the physics is that governs VDAC’s interaction with its protein partners, tubulin and αSyn, and how metabolite fluxes through VDAC are regulated by these interactions. We hope that answers to these questions will guide researchers in finding new potent endogenous regulators and inhibitors of VDAC and chart a path towards a long-overdue VDAC pharmacology.

## 2. How VDAC Permeability Is Regulated by Tubulin and α-Synuclein

**Figure 2:**
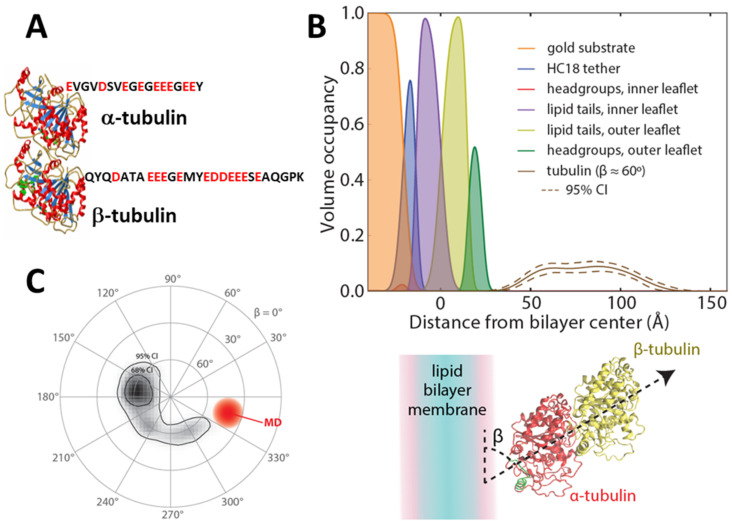
Structural properties of solubilized dimeric tubulin. (**A**) Crystal structure of tubulin (PDB ID: 1TUB) with the sequences of α1 and β3 C-terminal tails. (**B**,**C**) Orientation of peripherally membrane-bound tubulin as determined by neutron reflectometry and molecular dynamics simulations. (**B**) Volume occupancy of the molecular components of the lipid bilayer (filled curves) and membrane-bound tubulin described by Euler rotations of the crystal structure. The solid line shows the median volume occupancy of tubulin; the dashed lines show the 95% confidence interval. (**C**) Orientation plot of the Euler angles describing the rotation of the crystal structure to match the measured structure on the lipid surface. Both neutron reflectometry and molecular dynamics (MD) show an angle β between the dimer axis and the lipid surface of approximately 60°. CI = confidence interval. Adapted with permission from Hoogerheide et al., *Proc. Natl. Acad. Sci. USA* (2017). Copyright (2017) National Academy of Sciences.

Dimeric tubulin is an abundant cytosolic protein that constitutes a supply for polymerization into microtubules. Its structure is that of an α/β-heterodimer, water-soluble, compactly folded 110-kDa protein with a well-defined crystal structure [41] (Figure 2A). Each subunit has an unstructured CTT composed of 11–20 amino acids [42] that are exposed at the protein surface (Table 1). Both α- and β-tubulin CTTs are highly negatively charged and are essentially poly-Glu peptides. In microtubules, dimeric tubulin is assembled in such a way that the exposed, growing end of the microtubule is always the β-subunit. As a result, microtubule-targeting drugs (MTDs) used in chemotherapies bind exclusively to the β-subunit [43]. Until recently, any physiological role of unassembled, cytosol-solubilized dimeric tubulin, other than to serve as the building block for microtubules, remained unknown. Multiple tubulin genes in vertebrates result in an impressive variety of tubulin isotypes. There are seven α- and eight β-tubulin isotypes in humans, and the main difference between them is the length and the sequence of their CTTs [44] (Table 1). β-tubulin isotypes have the highest sequence variability and also different expression level in different tissues. For example, β2 constitutes up to 58% of the total β-tubulin in the brain [45] and β3 is found predominantly in neuronal cells and cancer cells of non-neuronal origin [46]. Interestingly, some of the lower eukaryotes have fewer tubulin genes. For example, yeast tubulin has only two α- and one β-tubulin isotypes [45]. Another intriguing feature of tubulin CTTs is that they possess most of the known tubulin post-translational modifications (PTM) [44]. The reason for such high molecular diversity of tubulin CTTs in vertebrates is not entirely clear (e.g., see discussion in [47]).

**Figure 3:**
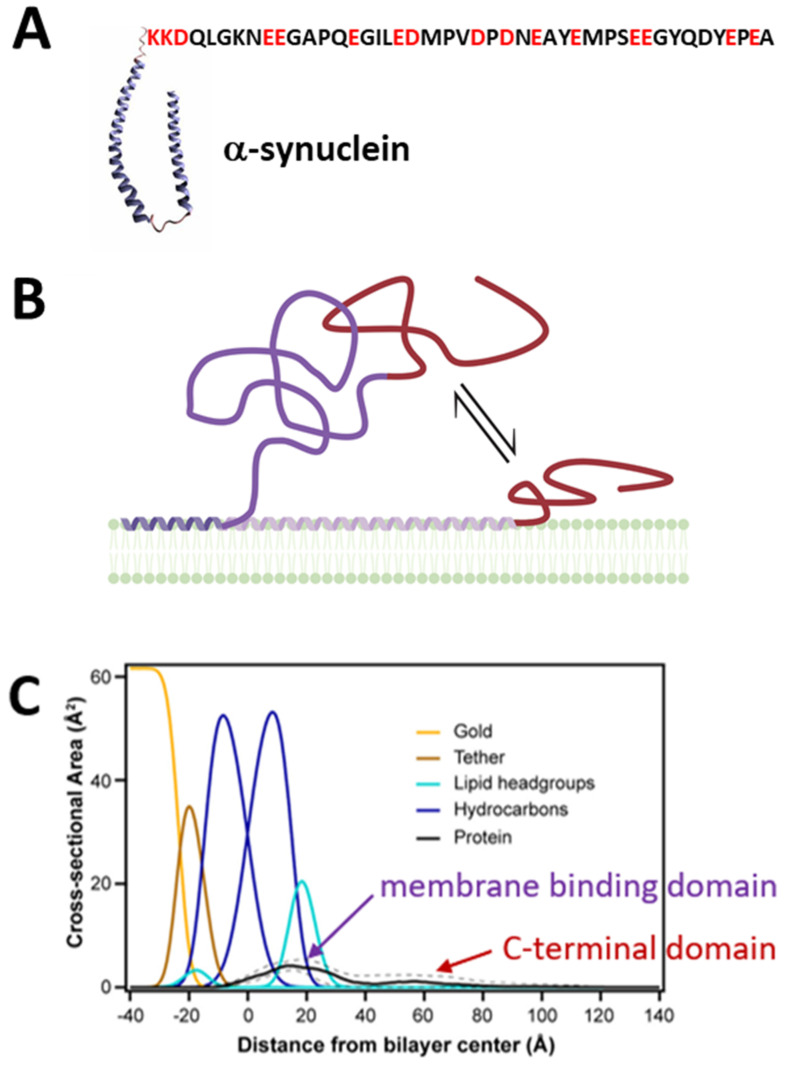
Structural properties of αSyn. (**A**) A schematic of membrane-bound αSyn with its helical N-terminal membrane-binding domain and its disordered C-terminal domain shown as the amino acid sequence (PDB ID: 1XQ8). (**B**) αSyn is peripherally membrane-bound and fluctuates between conformations where the C-terminal domain (red) is pinned close to, or floats further above, the membrane surface. Created with Biorender.com (accessed on 17 May 2021). (**C**) Volume occupancy of αSyn on lipid membrane surfaces is determined by neutron reflectometry in combination with selective perdeuteration. The membrane-binding domain is localized to the membrane, while the polyanionic C-terminal domain is broadly distributed, suggesting free-motion above the membrane surface. Solid and dashed lines show the median and 68% confidence interval, respectively, of the αSyn volume occupancy. Adapted with permission from Jiang et al., *J. Phys. Chem. Lett*. (2017). Copyright © 2016 American Chemical Society.

αSyn, by contrast, is a small, 14-kDa, 140 amino acid, intrinsically disordered protein (Figure 3A) under normal, solubilized conditions. αSyn is highly expressed in the central nervous system and constitutes up to 1% of the total cytosolic protein in normal brain cells [48]. While αSyn is abundant in the brain and presynaptic terminals of neurons, it is also found in smaller amounts in peripheral tissues, such as salivary glands, heart, and muscle [49]. αSyn is the major component of the Lewy bodies characteristically observed in the brains of Parkinson’s disease (PD) patients [50] where αSyn is present in a fibrillar form. Most studies have focused on the pathological role of αSyn aggregates, which are believed to be the main cause of αSyn toxicity in dopaminergic neurons in PD [51]. However, the precise role of αSyn in normal brain cells and in α-synucleinopathies, particularly in its monomeric form, remains largely mysterious.

### 2.1. Commonalities between Tubulin and α-Synuclein

Tubulin and αSyn, despite their clear differences, have three important commonalities. First, they are both found to be specifically associated with mitochondrial membranes. Tubulin was found bound to mitochondria from a wide range of cancerous and non-cancerous human cell lines [52,53,54]. Carré and coauthors estimated that mitochondria-associated tubulin represents ~2% of total cellular tubulin, but this value could vary significantly between cell lines from which mitochondria were isolated. In this study α- and β-tubulins were found at comparable levels in mitochondria, suggesting that mitochondrial tubulin is a heterodimer [53]. αSyn was found in association with both the MOM and mitochondrial inner membrane (MIM) in different cell models of dopaminergic neurons [55,56,57,58,59,60]. αSyn-induced mitochondrial dysfunction manifested itself in the impairment of the mitochondrial respiratory complexes, especially complex I, and in elevation of oxidative stress and inhibition of oxidative phosphorylation capacity [55,59,61]. While these αSyn effects on mitochondrial function are well-established in multiple studies during the last decade, several important aspects of αSyn involvement in mitochondrial bioenergetics and especially the precise role of monomeric αSyn and the molecular identity of the pathway for αSyn to cross the MOM, remained enigmatic until recently.

Second, both tubulin and αSyn belong to the family of the peripheral proteins, the water-soluble proteins that are transiently and reversibly associated with cellular and model membranes under certain conditions [62]. Tubulin binding to liposome membranes was reported in the early 1980s (for an exhaustive review see [63]). It took another three decades before a detailed structural picture of tubulin on a MOM-mimicking lipid membrane surface was obtained [64] using neutron reflectometry (NR). NR provides low-resolution structures of biological molecules and assemblies at solid-liquid interfaces [65]. In these experiments, a tethered bilayer lipid membrane (tBLM) was formed on a thin film of gold and exposed to a solution of dimeric tubulin. The reflectivity of the bilayer-containing interface to neutrons of varying incident angle reports on the structure of the solid-liquid interface containing the tBLM. Neutrons scatter from the nuclei of the target materials and are thus sensitive to the isotopic composition of the materials; as a result, neutrons have a contrast between lipid acyl chains, headgroups, and protein. In addition, the exchange of water for heavy water in the NR sample cell, or contrast variation, provides additional contrast and allows unambiguous determination of the underlying interfacial structure [66]. Variation in the morphological parameters of the bilayer and any associated proteins, such as bilayer thickness or the protein penetration depth or orientation, change the interfacial reflectivity. Measurements of these parameters involve optimizing a model of the interfacial composition to the experimentally determined neutron reflectivity [67]. The result of such optimization for tubulin on a 1:1 DOPC:DOPE bilayer is shown in Figure 2B; each molecular component of the tethered bilayer is shown by the filled curves, while the protein density is shown as the solid line (dashed lines are 95% confidence intervals). These measurements conclusively show that tubulin binds peripherally to the lipid membranes. The protein density profile corresponds to an orientation of the tubulin dimer axis relative to the membrane surface of approximately 60° (Figure 2C). In combination with molecular dynamics (MD) simulations, α-tubulin’s amphipathic helix H10 was identified as the likely membrane-binding domain. Other biophysical techniques revealed that tubulin binding is dependent on membrane lipid composition, with a strong preference for non-lamellar lipids with phosphatidylethanolamine (PE) headgroups over phosphatidylcholine (PC) [64,68].

In contrast to the sparse amount of works on tubulin-membrane binding, an impressive number of biophysical studies have been devoted to the interaction of αSyn with membranes [69,70,71,72,73]. Such high interest is understandable considering the established toxicity of αSyn fibrils in vivo and was inspired by the distinctive ability of lipid membranes to catalyze αSyn structuring and aggregation on their surface (for comprehensive reviews see [74,75,76]). Although disordered in bulk media, αSyn adopts secondary structure with a different helical content of its N-terminal domain depending on the lipid composition [69,70,71,74] and preferential binding to anionic and non-lamellar lipids, such as PE [77]. While the amphipathic N-terminal domain forms a structured membrane-binding domain on these membrane surfaces, the polyanionic CTT remains disordered [78]. Multiple binding conformations, differentiated by the amount of the amphipathic N-terminal domain in contact with the membrane surface, have also been reported [69] (Figure 3B). On membranes of other compositions, such as PC, the N-terminal domain also remains disordered upon membrane binding [79]. Consistent with these results, NR studies have confirmed the peripheral nature of αSyn membrane binding and shown protein density both at and away from the membrane surface (Figure 3C). Domain-specific deuteration of the N-terminal domain and/or the CTT allows identification of the N-terminal domain as responsible for membrane binding, while the CTT floats freely above the membrane surface [80].

Third, both tubulin and αSyn contain a disordered, highly polyanionic CTT (Table 1, Figure 2 and Figure 3). In the case of tubulin, both subunits present the CTT to the lipid surface [64]. In the following section, we will show that these features are required for measurable interaction with VDAC.

### 2.2. Role of the Disordered CTT

**Figure 4:**
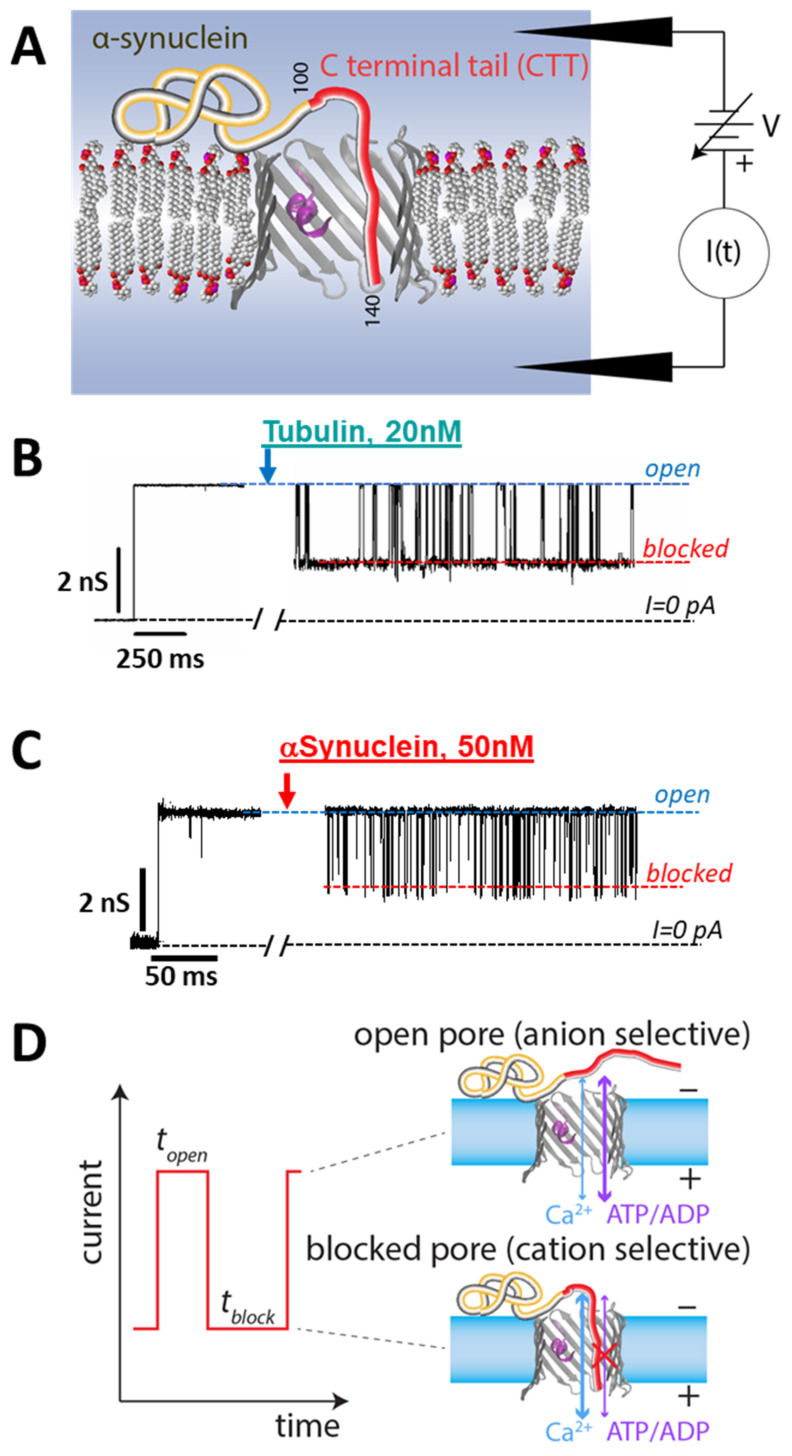
VDAC reconstitution measurements. (**A**) Experimental schematic. (**B**,**C**) A single VDAC channel is reconstituted into a lipid membrane; the inhibitor is then added and its effect on the transmembrane current monitored. Both tubulin (**B**) and αSyn (**C**) induce transient reductions of the VDAC absolute conductance. VDAC isolated from rat liver mitochondria was reconstituted into a planar lipid membrane of diphytanoyl-phosphatidylcholine bathed in an aqueous solution of 1 M KCl buffered at pH 7.4 with 5 mM HEPES (M = mol/L). The current records were taken in the presence of 20 nM wild-type tubulin (**B**) and 50 nM αSyn (**C**) at −27.5 mV of applied voltage and are shown as absolute values for clarity. Traces in (**B**,**C**) are adapted from Rostovtseva et al., *J. Biol. Chem*. (2018) (**B**) and from Hoogerheide et al., *Nanoscale* (2020), reproduced by permission of The Royal Society of Chemistry (**C**). For clarity of presentation, the records were smoothed with a 1 kHz lowpass Bessel digital filter using Clampfit 10.7. (**D**) Each interaction “event” is characterized by an onset time *t_open_* and a blockage time *t_block_*. The open and blocked states have opposite permeabilities to metabolites (e.g., ADP/ATP) and calcium ions.

When αSyn or dimeric tubulin are added to the bulk solutions surrounding a planar lipid membrane (PLM) with reconstituted VDAC (a schematic of the PLM system is shown in Figure 4A), they both induce characteristic fast blockages (on the 0.1 ms to 1000 ms time scale) of channel conductance (Figure 4B,C). In these VDAC reconstitution experiments, a single VDAC channel spans a PLM that separates and electrically isolates two buffer-filled compartments [7]. The ionic current through the VDAC channel is generated by an applied transmembrane potential and is continuously recorded. Without inhibitor addition, VDAC conductance is quite steady at low applied voltages of up to about 40 mV, depending on experimental conditions such as medium pH and lipid composition [7]. Above this potential, long (10 s to 100 s) voltage-induced gating transitions to gated states begin to occur; these are relatively rare and much longer-lived than inhibitor-induced blockages. Single-channel recordings of VDAC yield strikingly similar fluctuations of channel conductance between the uniquely open and blocked states induced by the addition of either dimeric tubulin or αSyn at nanomolar concentrations to the membrane-bathing buffer solution (Figure 4B,C). The conductance of the open state in the presence of either inhibitor remains the same as in the control, and the conductance of the blocked state is ~0.4 that of the open state conductance. For illustrative purposes, the current traces in Figure 4B,C were filtered to 1 kHz, but a larger bandwidth was used for the analysis of current fluctuations (see the detailed description in [81]). Such behavior is in striking contrast to the typical VDAC voltage-induced gating which is characterized by a variety of gated states [82,83]. The number of blockage events induced by tubulin or αSyn increases with the blocker protein’s bulk concentration [32,82]. 

Two important experimental observations lead us to propose that anionic CTTs of either inhibitor are the domains responsible for the reversible blockages of the VDAC pore. First, both tubulin and αSyn block VDAC only when a negative potential is applied from the side of their addition [32,82]. When the sign of the potential is reversed, no blockage events are observed, and the channel open state conductance is as steady as without inhibitor addition. This is in contrast with voltage-induced gating, which occurs at either voltage polarity. Second, tubulin with proteolytically cleaved CTTs does not induce VDAC blockages up to micromolar concentrations [31]. Likewise, αSyn truncated at residue 115, such that half the CTT is removed, induces ~10^4^ fewer blockage events than the full-length αSyn [32].

For both proteins, increased applied negative voltage keeps the anionic CTT inside the pore longer. This notion is supported by experiments that show that the blockage time—the time when the channel is blocked by tubulin or αSyn CTT (*t_block_* in Figure 4D)—is highly voltage-dependent and increases exponentially with the applied voltage for both inhibitors. This feature will be discussed in detail in Section 3.

Finally, evidence that the CTT of tubulin or αSyn is captured inside the pore, driven there by the negative voltage, was obtained in experiments where VDAC was reconstituted in a salt gradient. It was shown that the tubulin-blocked state has reversed, cationic selectivity, in comparison to the anion-selective open state [84]. This arises because the highly negatively charged tubulin CTT, by entering a net positive pore, reverses the net charge of the pore interior. The selectivity of the αSyn-blocked state is more complex, with the selectivity of the blocked state depending on which part of the αSyn molecule is trapped inside the pore at a given time [85,86]. On average, the selectivity of the αSyn-blocked state is also reversed, but it is less cation-selective than that of the tubulin-blocked state.

### 2.3. Role of the Membrane Anchoring Domain

The CTT alone is not sufficient to produce long-lived VDAC blockages. Synthetic peptides of α- and β-tubulin CTTs or peptides of the 45 amino acid long CTT of αSyn, for example, do not measurably block VDAC [31,32]. This suggests that the CTT should be attached to an “anchor” that localizes the CTT near the channel entrance and prevents free translocation through the pore, which occurs at time scales too fast to be experimentally observed [85]. For αSyn, this anchor is the N-terminal membrane-binding domain. For tubulin, it is the highly conserved amphipathic binding motif on the α-subunit [64].

A natural prediction is then that the membrane lipid composition is an important factor in the interaction of both tubulin and αSyn with reconstituted VDAC. This has been experimentally verified for both tubulin [64,68] and αSyn [87,88,89]. For both proteins, the proposed membrane-binding mechanism is the absorption of the binding helix into the lipid headgroup region governed by electrostatic and hydrophobic interactions, explaining the preference of both inhibitors for PE headgroups [64,68,87,88]. These features identify tubulin and αSyn as amphipathic proteins that sense lipid-packing stress and charge without involving the specific protein-lipid interactions observed for other membrane-active proteins such as lipases [90]. While amphipathicity may be common to these two mitochondria-targeting proteins, in principle any membrane-binding modality may serve as a membrane anchor.

### 2.4. A General Model of VDAC Inhibitor

**Figure 5:**
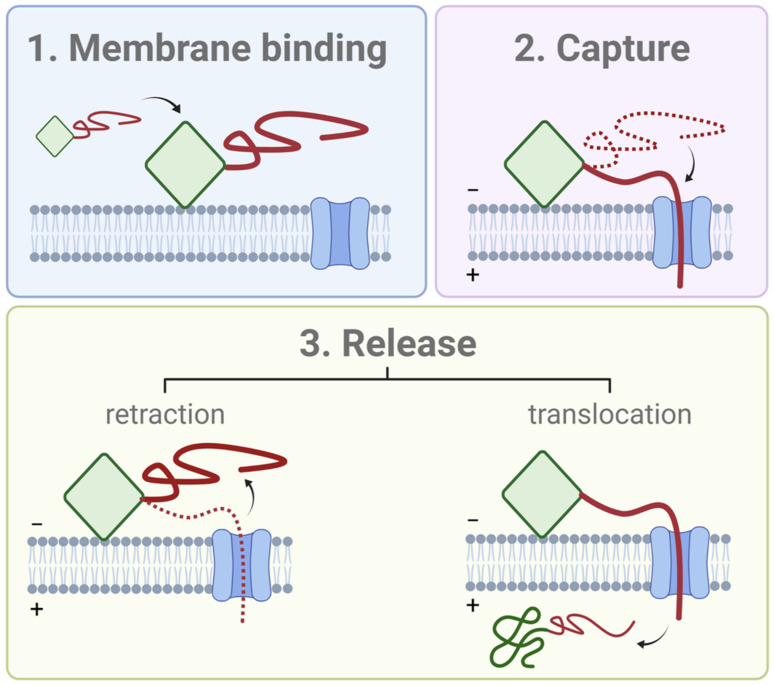
The general model of the structural features of a VDAC inhibitor and its interaction with a voltage-biased VDAC channel. The inhibitor has two domains: a membrane-binding domain (green), which can be structured or not, and a polyanionic, disordered domain (red). Interaction with VDAC begins with (1) recruitment of the inhibitor to the VDAC-containing membrane. (2) The polyanionic domain is captured into the VDAC pore by the transmembrane potential. Finally, (3) the inhibitor is released either by a retraction from the channel or translocation of the entire (unraveled) inhibitor. Created with Biorender.com (accessed on 3 May 2021).

Based on these data, a model of a general VDAC inhibitor is proposed, where the inhibitor requires two distinct parts: the polyanionic disordered pore-blocking domain, which is drawn into the VDAC pore by the transmembrane potential; and the membrane-bound amphipathic domain which holds it at the membrane surface, hindering complete translocation of the inhibitor through the channel (Figure 5). The VDAC inhibition process proceeds, first, by the recruitment of the inhibitor by the VDAC-containing membrane; second, by the capture of the polyanionic domain into the VDAC pore; and third, release by either retraction of the polyanionic domain to the same side of the channel from where it entered, or translocation (in the case of αSyn) of the entire protein to the opposite side of the channel. This model of VDAC inhibition provides a unified description of how such different proteins as tubulin and αSyn induce qualitatively similar blockages of VDAC. Importantly, the model is quite general and does not require any specific interactions between the VDAC and inhibitor. The lipid membrane plays a crucial role in recruiting the inhibitor to the membrane surface, thus concentrating it in the vicinity of VDAC; moreover, membrane composition is an effective modulator of the availability of the inhibitor for capture.

## 3. Quantitative Analysis of VDAC Regulation by Tubulin and α-Synuclein

To understand how a measurable interaction can occur at nanomolar solution concentrations of tubulin or αSyn without specific binding between two proteins, one needs a quantitative examination of the physics that governs VDAC’s blockage by its protein partners. The efficacy of a VDAC inhibitor is determined primarily by its availability to the VDAC channel and the energetics of its interaction with VDAC. In this section, we focus on the qualitative features of the latter, as revealed by extensive in vitro experiments involving both tubulin and αSyn. The single-molecule nature of the experiments shown schematically in Figure 4D enables a detailed study of the interactions of thousands of individual tubulin or αSyn molecules with a single VDAC channel. Each interaction is colloquially known as an “event”. In these experiments, the free energies of interaction can be determined by the voltage dependence of two experimental observables: the rate at which the inhibitor is captured into the VDAC pore (characterized by the inverse mean of the time elapsed between the consecutive events, *t_open_*), and the rate at which it is released (characterized by the inverse mean of the time elapsed between the capture and release, *t_block_*). The most effective blockers have a *fast rate of capture* and *a slow rate of release*, such that the VDAC channel spends most of the time occupied by the inhibitor.

**Figure 6:**
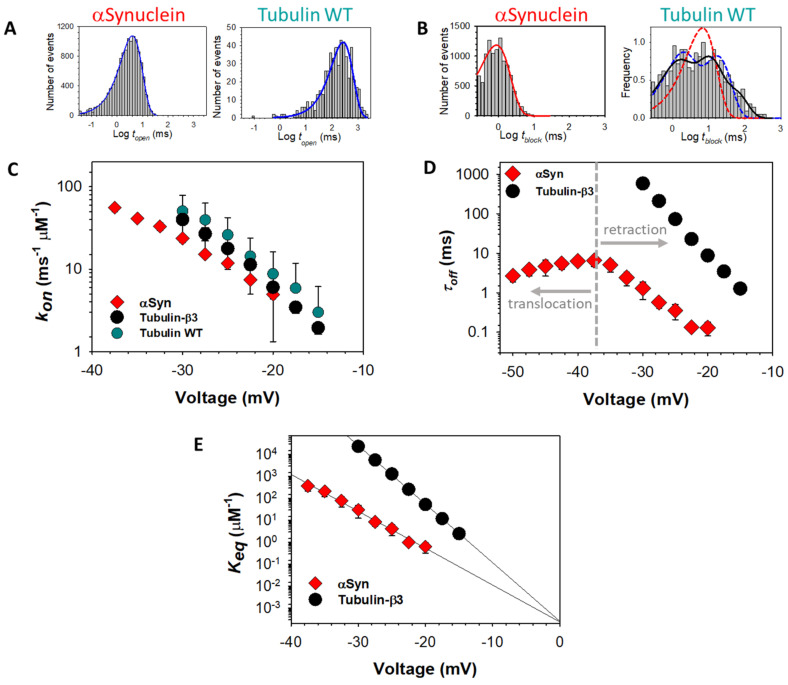
Distributions of (**A**) open and (**B**) blocked state times for VDAC exposed to tubulin and αSyn. (**C**) The capture rate is exponentially dependent on the transmembrane potential and is similar for both inhibitors. (**D**) The release rate is also exponential in voltage for tubulin and αSyn at low voltages but is more complex for αSyn at higher voltages, indicating the onset of αSyn translocation through VDAC. (**E**) The equilibrium constant, extrapolated to zero voltage, reveals the entropy of insertion, which is the same for the C-terminal domains of tubulin and αSyn and points to a common mechanism of interaction. VDAC isolated from rat liver mitochondria was reconstituted into PLM made of diphytanoyl-phosphatidylcholine in membrane-bathing 1 M KCl solution buffered with 5 mM HEPES at pH 7.4. Distributions of open (**A**) and blocked (**B**) times were obtained in the presence of 50 nM αSyn and 20 nM wild-type tubulin at −25 mV (**A**) and −30 mV (**B**) of applied voltage. Data were adapted from Rostovtseva et al., *J. Biol. Chem*. (2018) and Rostovtseva et al., *J. Biol. Chem*. (2015) and used in newly generated figures (**C**–**E**). Data points denote mean values from independent repeated experiments; error bars show the 68% confidence interval of the mean.

These rates are determined experimentally from the distributions of the two times associated with each event (Figure 4D). When a histogram of either of these times is built on a semi-log scale as log-binned distributions and is described by a single exponent (Figure 6A,B), the characteristic exponential times corresponding to distributions of *t_open_* and *t_block_* are simply equal to the averages of all the individual *t_open_* and *t_block_* observations, respectively, and are denoted $\tau_{on}$ and $\tau_{off}$. In practice, the distributions of *t_open_* are always described by a single exponent with characteristic capture time $\tau_{on}$ for both tubulin and αSyn (Figure 6A). However, the distributions of *t_block_* often require multi-exponential functions for their description, denoting the underlying heterogeneity in the captured polypeptide strands (Figure 6B, *right panel*). These features, and their physical ramifications, will be explored in the following subsections.

### 3.1. Release Rate

The distributions of *t_block_*, which correspond to the times elapsed before the release of each docked molecule from the pore, are usually complex and highlight the differences between tubulin and αSyn. For tubulin, at least two exponential components are required to describe the *t_block_* distribution (Figure 6B, right panel). A release time distribution that is wider than a single exponential distribution suggests heterogeneity; indeed, the wide *t_block_* distribution is characteristic for the wild-type mammalian tubulin isolated from the natural sources (such as the brain) that consists of a mixture of multiple α- and β-isotypes, i.e., a mixture of CTTs of different length and charge (Table 1). When recombinant yeast α/β-tubulin constructs with one, either α- or β-CTT, were used in experiments with reconstituted VDAC, the *t_block_* distributions were satisfactorily described by single-exponential functions [91]. The highly homogeneous recombinant tubulin constructs, containing two CTTs such as those from α1 and β3 that differ in length and charge (Table 1), induced two well time-resolved populations of *t_block_* [91], clearly indicating that both α- and β-CTTs could be captured by VDAC pore. This observation confirms the NR and MD study that showed a membrane-bound conformation of tubulin that presented the CTTs of both subunits to the membrane surface [64].

**Figure 7:**
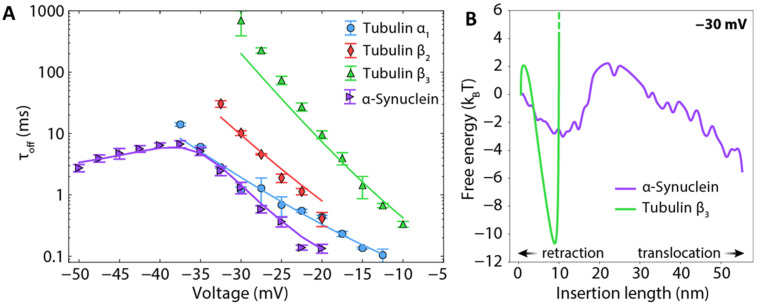
Energy landscape modeling of release rates of the CTTs of various tubulin isotypes and wild-type αSyn. (**A**) Experimental data (markers) and energy landscape model (solid lines) show excellent agreement between the model and experiment with few free parameters. Each data point is a mean of 3–5 independent experiments, and error bars represent the standard error of the mean. Data were adapted from Rostovtseva et al., *J. Biol. Chem*. (2018). (**B**) Free energy profiles underlying the curves in (**A**). The reaction coordinate is the length of the polypeptide strand that has been inserted into the VDAC channel.

Strikingly, individual tubulin CTTs block the VDAC pore with *τ_off_* that differs by orders of magnitude and is closely related to the total charge of the particular tubulin CTT (Table 1 and Figure 7A). However, prediction of the release rate based on total charge fails in the case of αSyn, which has a total charge similar to β3-tubulin, but 100 times faster release (Figure 6D).

The anomalously fast release of αSyn relative to tubulin is not the only difference. For intrinsically disordered αSyn, the whole molecule can, in principle, translocate through the channel because a single polypeptide strand fits comfortably into the ~2.5–2.7 nm diameter VDAC pore [92]. In contrast to the tubulin-induced blockages, *τ_off_* of αSyn-induced blockages shows a clear biphasic behavior: blockage time increases exponentially up to a certain voltage, V*, (~−40 mV for conditions as in Figure 6D) and then markedly decreases at higher voltages [32]. Direct measurements of the translocation probability of αSyn [86] unequivocally demonstrate that the decrease of dwell time at *V* > |V*| is indicative of the onset of a translocation regime, where the applied voltage drives the entire αSyn molecule through the pore. Therefore, under certain conditions, such as the applied potential, lipid composition, and ionic strength of buffer solution, αSyn can either block VDAC in a qualitatively similar manner to tubulin or translocate through the channel (Figure 5). Tubulin, due to its large globular component, cannot physically translocate without unfolding, and thus no V* is observed; the tubulin CTT is released only by retraction (Figure 5).

Importantly, it has been extensively shown [81,85,86,87,91] that the release dynamics can be described by stochastic equations of motion that account for the sequence-specific electrostatic free energy, entropy of confinement, and membrane dissociation energy without appealing to direct biochemical interactions between the VDAC pore and the tubulin and αSyn CTTs. This “energy landscape modeling” of the threading of polypeptides into a nanopore is aided by a natural one-dimensional “reaction coordinate”, i.e., the length of a polypeptide that has passed through the pore. With the appropriate constraints, these equations describe equally well the dynamics of both tubulin and αSyn, for which Figure 7B shows the exemplary free energy profiles underlying the theoretical predictions in Figure 7A. Importantly, this allows the prediction of the behavior of any protein that has the characteristics of a VDAC inhibitor; a prediction tool that solves the underlying dynamical equations for any protein sequence is available online [93].

Energy landscape modeling reveals that the structure and sequence of the captured strand determine the number of elementary charges that can move through the VDAC pore before its motion is arrested by the membrane anchor. For both tubulin and αSyn, this is a large number that ranges from 7 to 14, providing exquisite sensitivity of the free energy barrier of retraction to the transmembrane potential. Thus, for the various tubulin isotypes, which vary drastically in the length and number of charged residues of the CTTs (Table 1), the rate of release varies by many orders of magnitude (Figure 7A) [91]. In addition to the CTT length and net charge, the distribution of charges in the CTTs also has a strong effect; the effect of charged residues near the ends of the CTTs is offset by the strength of the entropic potential in this region [81]. These results also explain the anomalously fast release rate of αSyn relative to β3-tubulin, despite similar total charge; the entire αSyn CTT cannot pass through the VDAC pore before being arrested by the membrane anchor.

Similarly to tubulin, distributions of *t_block_* for αSyn, in general, require more than a single-exponential function to describe. However, the source of heterogeneity is not in the amino acid sequence of the CTT, as it is for tubulin. On membranes of certain compositions (neutral and positively charged lipids), VDAC captures a high fraction of αSyn molecules in which the C-terminal domain floats freely above the membrane surface (Figure 3B). Once captured, the entire complement of polyanionic residues in the C-terminal domains of such molecules pass through the pore, leading to longer residence times. By contrast, molecules in conformations that pin the C-terminal domain near the membrane surface have shorter residence times, as fewer charged residues are captured by the pore and thus influenced by the electric field. The electric field also applies a continuous destabilizing force to such molecules, increasing the likelihood of membrane detachment and eventual translocation [87,89].

Notably, energy landscape modeling assumes a mostly static VDAC channel conformation and therefore does not account for the dynamics of the VDAC molecule [94,95]. There is no evidence from the experimental data that these dynamics play a significant role, perhaps because the millisecond duration of each blockage event tends to be much longer than, and thus averages, the sub-microsecond barrel dynamics. Furthermore, very similar qualitative behavior is observed with a much less dynamic β-barrel channel, α-hemolysin [89,96].

### 3.2. Capture Rate

The capture rate can be quantified by the on-rate constant of VDAC’s interaction with an inhibitor, *k_on_*, which is inversely proportional to *τ_on_* and normalized to the bulk concentration of inhibitor, [*C*], i.e., *k_on_* = 1/τ_on_ [*C*]. The on-rate is an exponential function of the applied voltage; notably, the slope of the *k_on_* voltage dependence and *k_on_* values obtained under the same experimental conditions, such as lipid membrane composition, medium salt concentration, and pH, were found to be very similar for αSyn and tubulin (Figure 6C).

When combined with the release rate dynamics, the capture rate allows estimation of the free energy of confinement of αSyn in the VDAC channel to be approximately 13 *k_B_T* [85]. Similarly, insertion of a second *uncharged* polypeptide strand into VDAC requires additional free energy of 5 *k_B_T*, while insertion of a second *charged* polypeptide strand was never observed. This is consistent with the observed reversal of the channel selectivity upon insertion of a single charged strand [85,86]. It is thus expected that capture of single strands is much more favorable than that of disordered polyanionic loops and that at least some transmembrane potential is required for a measurable capture rate. Note that the energy of confinement for tubulin in the absence of voltage is identical to that of αSyn, as can be directly observed from extrapolation of the equilibrium constant $K_{eq}=k_{on}\tau_{off}$ to zero voltage (Figure 6E). This important observation further demonstrates that tubulin and αSyn alter VDAC’s transport properties by the same fundamental mechanism shown in Figure 5.

Rather counterintuitively, but of potential significance, we found that the conformation of αSyn on the membrane surface (Figure 3B) can determine the probability of capture by the VDAC pore [87]. As discussed above, αSyn is intrinsically disordered in solution but can adopt an ensemble of membrane-bound conformations [69]. These αSyn conformations are known to be strongly dependent on membrane lipid composition [69,74,79]. Analysis of the lipid-dependent dynamics of the αSyn-VDAC interaction suggests that it is defined by the conformational ensemble of membrane-bound αSyn molecules [87]. In addition, conformations that pin the beginning of the C-terminal tail (a region between the non-amyloid-component, NAC, and CTT domains) of αSyn near the membrane surface are readily captured by VDAC but are also readily released; by contrast, conformations that allow the C-terminal tail to float freely above the membrane surface are captured at a lower rate (Figure 3B) [89]. Thus, lipid composition may be yet another means by which cells can regulate the VDAC-αSyn interaction.

### 3.3. Specificity of the Interaction with the VDAC Pore

The energy landscape models do not include a direct interaction between the anion-selective (i.e., net positively charged) VDAC pore and the captured polyanionic strand. However, from purely electrostatic considerations such an interaction is expected to exist. While the details depend on the polyanionic charge density of the strand, which differs for tubulin and αSyn (Table 1), the magnitude of this effect can be estimated from the existing experimental data. Two independent measurements suggest that the magnitude of this interaction is several *k_B_T*. First, tubulin is captured about 4 times less readily at high electrolyte concentrations, where this interaction is screened [97], suggesting the loss of an interaction energy of about $\ln4\approx 1.4\;k_{B}T$ (including an unknown contribution from changes in the density of membrane-bound tubulin with salt concentration). Second, the partitioning between the neutral and charged regions of αSyn can be directly measured under transmembrane electrolyte gradients and also shows that occupancy by the charged region is favored [86]; extrapolation of this weakly voltage-dependent partitioning curve to zero potential (Figure 6E) yields 3.4 *k_B_T*. On average, then, the magnitude of the electrostatic interaction between the polyanionic charged strand and the cationic VDAC lumen is expected to be on the order of (2 to 3) *k_B_T*. Note that this interaction is thus a relatively small correction to the membrane dissociation energy in the energy landscape modeling of αSyn, but can have more than an order of magnitude effect on capture rates. Finally, the capture of αSyn into the cation-selective Tom40 channel has not been observed [98], supporting the idea that electrostatic interactions between the channel and the polyanionic inhibitor strand significantly alter the rate of capture.

### 3.4. Modeling Post-Translational Modifications In Vitro

**Figure 8:**
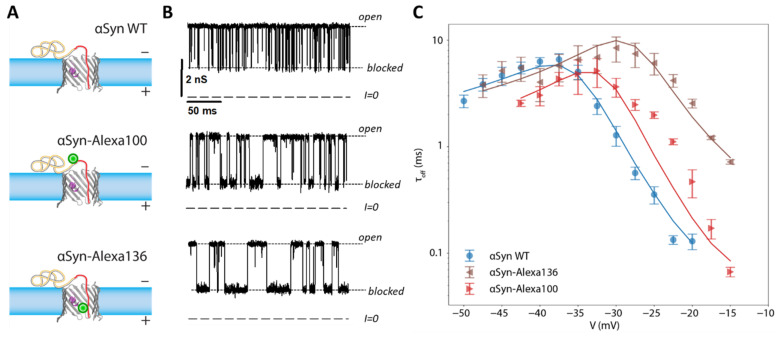
Effect of a mimic post-translational modification on the interaction of αSyn with VDAC. (**A**) Schematic of wild-type αSyn and two constructs with a bulky Alexa Fluor 488 side chain, green circle, attached at residues 100 and 136 (at the beginning and the end of the CTT). (**B**) The dynamics of interaction with VDAC are visually altered by the modifications, as witnessed by the time record of channel conductance. (**C**) Effect of CTT modifications on blockage time (i.e., the inverse release rate). Solid lines are results of energy landscape modeling. Each data point is a mean of repeated independent experiments, and error bars represent the standard error of the mean. Adapted with permission from Hoogerheide et al., *Nanoscale* (2020). Reproduced by permission of The Royal Society of Chemistry.

An illustration of the effects of PTMs is shown in the model case where Alexa Fluor 488 was covalently attached to αSyn (Figure 8A,B). The relatively bulky sidechain of Alexa Fluor 488 introduces an entropic barrier to capture and release as well as adding two negative charges to the αSyn CTT. These modifications profoundly affect the release dynamics [81], leading to longer blockage times and an onset of translocation at lower voltages (Figure 8C).

These results can be generalized as follows: any PTM that adds a negative charge to the CTT will increase an inhibitor’s response to voltage, decrease release rates, and enhance its stability in the VDAC channel. On the other hand, protruding sidechains will introduce an entropic barrier that is expected to decrease both its capture and release rates and thus do not in general have a strong effect on the overall inhibition effectiveness. In the most extreme cases, where an inhibitor like tubulin has a large globular domain, translocation of the inhibitor is completely disallowed.

### 3.5. Effect of Salt Concentration

In vitro data are routinely obtained in the relatively high salt concentration of 1 M KCl to aid VDAC reconstitution into planar membranes and improve the signal-to-noise ratio of the electrophysiology measurements. Nonetheless, a substantial body of measurements performed at 150 mM KCl show that nearly all effects obtained in the presence of tubulin or αSyn are more pronounced [88,97]. This result is expected given that at high salt concentrations electrostatic interactions are largely screened.

## 4. Physiological Implications of α-Synuclein and Tubulin Interaction with VDAC: Similarities and Differences

### 4.1. Metabolite Transport

VDAC is a metabolite channel; thus, the main purpose of studying its interactions with other proteins is, most probably, to understand how they could affect the transport of adenosine phosphates and calcium through the channel and consequently, how docked proteins might regulate mitochondrial function. The large body of structural, functional, and computational research on VDAC probes its open state, which can support the necessary level of ATP flux across MOM [17]. It was shown that the adenosine phosphates weakly bind inside the pore with a millimolar binding affinity [99,100]. Such binding boosts their translocation according to a model where the loss of solute entropy due to confinement by the pore is compensated by solute-pore attraction [101,102]. Recent computational studies on VDAC1 confirmed the existence of ATP-binding sites, some of which are located on cationic residues Lys12 and Lys20 [103] of the N-terminal domain, and defined multiple permeation pathways for ATP [104]. All these data confirm that VDAC in its open state effectively translocates ATP and other polyanionic mitochondrial metabolites.

The question is then whether VDAC remains permeable to ATP when it is blocked by an inhibitor or interacts with a protein partner. A combination of in vitro channel experiments with MD simulations unambiguously demonstrated that ATP is excluded from the tubulin-blocked state [84,103]. The presence of the negatively charged tubulin CTT in the pore creates a steric and electrostatic barrier for ATP translocation of 2 kcal/mol to 3 kcal/mol (8 kJ/mol to 13 kJ/mol). The basic residues of VDAC’s N-terminal domain, Arg15, Lys20, Lys12, and Lys32, form stable salt bridges with the acidic CTT of a bound α-tubulin [103]. Some of these basic residues were found to participate in the formation of a high-affinity ATP binding site inside the VDAC pore and therefore to disrupt ATP transport through the tubulin-blocked state of VDAC. These in vitro and in silico results suggest a disruption of ATP/ADP fluxes through the tubulin-VDAC complex that could lead to a decrease of mitochondrial potential in vivo. Indeed, these predictions were first confirmed in experiments with isolated mitochondria [31,105] and then, by Maldonado and coauthors, in experiments with human hepatoma HepG2 cells where the cytosolic pool of free dimeric tubulin was modulated by MTDs [106,107]. It was shown that those MTDs that stimulate microtubule depolymerization and consequently the increase of cellular free tubulin (such as colchicine, rotenone, and nocodazole) caused a decrease of mitochondrial potential in HepG2 cells, and MTDs that stabilize microtubule and decrease free tubulin (such as paclitaxel) caused an increase of potential [106]. However, considering that the mitochondrial potential could be modulated by MTDs through several pathways unrelated to VDAC [108,109], such as promoting apoptosis by inducing MOM permeabilization in neuroblastoma cells and isolated mitochondria [110], inhibiting mitochondria biogenesis [111], or inducing mitochondrial network fragmentation [112], the genetic evidence of VDAC’s involvement in the modulation of the mitochondrial potential by MTDs was required. Such evidence was provided by the same group using the siRNA knockdown of individual VDAC isoforms in HepG2 cells [107]. They unambiguously demonstrated that all three VDACs, though to different extents, contribute to maintaining the mitochondrial membrane potential in response to cell treatment by MTDs. Later, β3-tubulin and VDAC were confirmed to be in close proximity in differentiated neuroblastoma cells using a proximity ligand assay (PLA) [91,113]. Together, these studies point to the functional importance of the VDAC-tubulin interaction for regulating mitochondrial respiration in intact cells. One of the intriguing implications of VDAC regulation by tubulin is its coupling with the Warburg-type aerobic glycolysis characteristic of many tumor cells [114], where the VDAC-tubulin complex may play a role of “glycolytic switch” in cells towards aerobic glycolysis or oxidative phosphorylation [82,115]. Notably, β3-tubulin, which is found exclusively in neuronal cells and in large numbers of cancer cells of non-neuronal origin [46] and which induces the most effective VDAC blockage in comparison with other studied tubulin isotypes and αSyn in reconstitution experiments (Figure 7A), has also been associated with tumor development and aggressiveness. The well-known resistance of β3-tubulin isotype to chemotherapy in tumors with poor prognosis [116,117] is the main reason for high interest in the cancer research field to this isotype.

The disordered nature of αSyn has, to date, prevented computational studies of the αSyn-VDAC complex. We have established, however, that αSyn interacts with VDAC by the same mechanism as tubulin; thus, we predict similar disruption of ATP/ADP fluxes through VDAC blocked by αSyn.

### 4.2. Calcium Transport

VDAC’s involvement in the transport and regulation of mitochondrial calcium is another interesting question to address with respect to VDAC’s interaction with its inhibitors, because VDAC is known to be a part of multi-protein complexes connecting VDAC with calcium channels in other organelles tightly associated with MOM, such as endoplasmic and sarcoplasmic reticulum [118,119,120] and lysosomes [121]. These protein complexes form calcium microdomains to ensure the rapid transfer of calcium to mitochondria from the intracellular calcium stores. Using VDAC reconstitution experiments, it was initially shown by Colombini’s group that VDAC might regulate calcium fluxes to and from mitochondria by voltage gating [15]. More recently, our group has demonstrated that calcium flux through VDAC is greatly facilitated by αSyn [122]. These experiments were performed on VDAC reconstituted in a membrane subjected to a CaCl_2_ salt gradient that allows measurement of VDACs ionic selectivity in the open and αSyn-blocked states. From these measurements, the calcium currents through both states were calculated, showing that the calcium permeability of the blocked state is >10 times higher than that of the open state [122]. The high permeability to calcium in the presence of αSyn is due to the favorable electrostatic environment created by the anionic CTT inside the pore, which overcompensates the physical obstruction of the pore by an order of magnitude.

Since most tubulin CTTs have a higher negative charge density than CTT of αSyn (Table 1) and higher cation selectivity of the blocked state [84], we anticipate that tubulin-blocked VDAC will be even more permeable to calcium. Though this prediction still needs direct experimental support, it opens the intriguing possibility of the existence of other potent cytosolic regulators of calcium fluxes through VDAC.

### 4.3. (Patho)physiological Role of α-Synuclein Translocation

Considering the substantial level of endogenous αSyn in neurons [48], where it is routinely found in association with mitochondria [58,123,124,125], we can further speculate that under normal conditions αSyn regulates metabolite fluxes by reversibly blocking VDAC (Figure 4D). Under stress conditions manifested in synuclein overexpression, αSyn translocates through VDAC into the mitochondria, where it targets and impairs the complexes of the electron transport chain (ETC) in the MIM [57,59,123,124,126]. The damaging effect of αSyn on ETC complexes I [59,123,126] and especially V [57] has been consistently reported. Mitochondrial dysfunction induced by αSyn overexpression is evidenced by a decrease of the mitochondrial potential [98,123,127], a decrease in ATP production [128], and an increase of ROS [55,129], eventually leading to neuronal cell death (see a recent review [130]). Recently, using neuronally differentiated human cells overexpressing wild-type αSyn, our group showed that downregulation of VDAC1 with siRNA results in the prevention of αSyn colocalization with complex IV at the MIM, thus establishing VDAC1 as a pathway for αSyn translocation into mitochondria [98]. A question about the possible roles of the other two VDAC isoforms, VDAC2 and VDAC3, in αSyn-imposed regulation of ATP/ADP fluxes and its translocation into mitochondria remains open. Recent biophysical experiments on reconstituted VDAC3 isoform showed that αSyn blocks VDAC3 in a qualitatively similar manner to VDAC1, but with a few orders of magnitude reduced on-rates and different blocking times [131]. Importantly, it was not only shown that αSyn translocates through VDAC3 but that the translocation regime starts at about 10 mV lower potentials than those found for VDAC1. These results allow us to speculate that in cells the less efficient capture of αSyn by VDAC3 could result in VDAC3 remaining open for ATP/ADP transport, while at the same expression level of αSyn, VDAC1 could by mostly blocked by αSyn. αSyn’s affinity to VDAC2 is a subject of ongoing research in our lab.

The complexity of αSyn’s interaction with VDAC highlights the need for further studies on the cellular level. Nonetheless, it is reasonable to surmise that the (patho)physiological roles of monomeric αSyn originate from its interaction with VDACs. Future research will show how the proposed mechanism by which αSyn interacts with VDAC and enters mitochondria could explain its neurotoxicity and whether it could help in identifying neuroprotective drugs.

### 4.4. Physiological Role of Post-Translational Modifications

Although most of the PTM sites of αSyn belong to its N-terminal and NAC domains, multiple sites of phosphorylation, nitration, ubiquitination, and sumoylation are found in the C-terminal domain [132,133]. Small, uncharged PTMs (e.g., oxidation, nitration, dopamination) are unlikely to have a strong effect on the release dynamics (see Table 2, adapted from [81]). However, we expect that in vivo, phosphorylation of the αSyn CTT promotes αSyn translocation to the MIM, where it disrupts mitochondrial function. On the other hand, ubiquitination and sumoylation of αSyn should lead to the opposite outcome. The attachment of these globular proteins will prevent αSyn translocation through the pore, thus protecting mitochondria from αSyn toxicity.

Notably, almost all tubulin PTMs occur at its CTTs [42,44,47]. This especially concerns tubulin polyglutamylation, which is manifested through the formation of linear branches on the CTTs up to 21 glutamates [134]. The level of polyglutamylation in brain tubulin changes dramatically during development: polyglutamylation increases in β-tubulins starting from nonglutamylated β-isoforms in neonatal animals and reaching a high level of polyglutamylation in adult brains [44]. 3–6 glutamates per branch are typically found in tubulin purified from adult rodents’ brain tissue [135]. The branched CTT can interact with the VDAC pore in a variety of ways from increasing the capture probability and residence time to minimizing them, depending on the length, volume, and position of the charged branch on the tubulin CTT. Therefore, predicting the impact of tubulin polyglutamylation on its interaction with VDAC in vivo is difficult and requires further experimental study. (By contrast, polyglycylation, which increases the physical size of the tubulin CTT without increasing its total charge, changes only the entropic interaction of the tubulin CTT with VDAC and is unlikely to have a strong overall effect).

We anticipate that the effect of tubulin polyglutamylation on VDAC permeability and consequently on mitochondrial metabolism in the brain changes dramatically during development. αSyn also accumulates in the brain with age; it is thus tempting to speculate that in the brain, the role of nonglutamylated tubulin as a VDAC inhibitor in neonatal organisms could be passed on to αSyn in adults. Further research is necessary to confirm this conjecture. We believe that this newly suggested mechanism of PTM-induced regulation of VDAC-facilitated metabolite and calcium transport could give important clues to our understanding of the nature of the surprisingly multifunctional role of this relatively simple β-barrel mitochondrial protein.

By contrast to polyglutamylation and polyglycylation, phosphorylation is found exclusively on β3 and β4-tubulin CTTs [44]. Because phosphorylation adds two negative charges to the CTT without significantly changing its size, phosphorylated CTTs will respond even more strongly to transmembrane potential. Whether phosphorylation plays an important physiological role by this mechanism remains to be determined. Detyrosination/tyrosination is manifested in the removal or addition of the gene-encoded tyrosine at the end of α-tubulin CTT [44]. This process slightly alters the length of the CTT but does not affect the overall charge and is thus not expected to have a significant effect.

### 4.5. Implications for the Mitochondrial Outer Membrane Potential

The foregoing discussion demonstrates that, in the context of the mitochondrial outer membrane, the fundamental role of tubulin and αSyn appears to be to catalyze the closure of VDAC to anionic metabolites and its opening to calcium. Importantly, as shown schematically in Figure 1, in the presence of these cytosolic inhibitors, a much smaller transmembrane potential is required to have the same overall effect as voltage gating. At typical cytosolic concentrations of free tubulin of ~5 µM [132], for example, only about 10 mV of transmembrane potential is estimated to be required to achieve 50% closure of VDAC (Figure 6E). This value is of a similar magnitude to the ever-present Donnan potential [136] and may be enhanced by the mechanisms outlined by Lemeshko, who estimated that with mostly closed VDAC, the outer membrane potential could achieve values as high as 50 mV [22,23,24]. Because tubulin and αSyn close VDAC at much lower potentials, it is conceivable that a 50 mV potential difference may not be required in vivo but regardless is readily achievable.

**Table 2:** Post-translational modifications to polyanionic C-terminal domains of αSyn and tubulin. Predicted physiological effects are relative to unmodified αSyn or tubulin. Adapted with permission from Hoogerheide et al., *Nanoscale*. (2020). Reproduced by permission of The Royal Society of Chemistry.

PTM	CT Residues Affected	Effect on Residue Charge	Increase in Residue Volume	References	Predicted Physical Effects	Predicted Physiological Effects
α-Synuclein						
Phosphorylation	125, 129, 133, 136	−2	54 Å^3^	[137]	Higher translocation probability, longer dwell time at low voltages	Decreased metabolite flux, increased calcium flux, ECT impairment
Nitration	125, 133, 136	0	53 Å^3^	Molecular volume from MW and density of NO_2_ liquid	Minimal	Minimal
Ubiquitination/Sumoylation	96/96, 102	0/−5 (does not affect CTT properties)	11,130 Å^3^/14,890 Å^3^	[138]	Eliminates translocation, longer dwell time at high voltages	Reduced ECT impairment, decreased metabolite flux, increased calcium flux
Dopamination	125–129	0	150 Å^3^	[138,139]	Smaller capture rate, longer dwell time at low voltages	Minimal
Tubulin						
Polyglutamylation (N residues)	varies by isotype	−(N + 1)	N × 155 Å^3^	[138]	Higher capture rate, longer dwell time	Decreased metabolite flux, increased calcium flux
Polyglycylation (N residues)	varies by isotype	−1	N × 66 Å^3^	[138]	Smaller capture rate, longer dwell time	Minimal
Phosphorylation	437, 444 (β3) 441 (β4)	−2	54 Å^3^	[137]	Longer dwell time	Decreased metabolite flux, increased calcium flux
Tyrosination/ Detyrosination	Last residue on α-tubulin	0	by 204 Å^3^	[138]	Minimal	Minimal

## 5. Conclusions

VDAC regulates fluxes of metabolites and calcium across the MOM by transiently switching between the states in which its permeability for metabolites and calcium is reversed. As previously established, at sufficiently high transmembrane potentials, VDAC can achieve this voltage-induced switching, or gating, on its own. What we have now shown is that the abundant cytosolic proteins tubulin and αSyn can induce, in a highly voltage-dependent manner, conductance states with the same effect on metabolite and calcium fluxes as the voltage-gated states at much smaller transmembrane potentials. Both tubulin and αSyn share three common features: a membrane-binding domain; a polyanionic, disordered C-terminal domain; and a molecular conformation on lipid membranes that presents the C-terminal domain to the membrane surface and hence to membrane-embedded VDAC. The mechanism of their docking to VDAC is the same and seems to be quite general: it is recruitment to the VDAC-containing membrane followed by the capture of the C-terminal domain into the VDAC pore by the transmembrane potentials. This suggests a strategy for designing a general VDAC inhibitor and thus opens a long-sought avenue toward establishing VDAC pharmacology. Interactions with cytosolic proteins reviewed in this article allow us to expect that additional endogenous VDAC regulators will also be identified. Finally, the detailed physical model that describes the motion of the C-terminal domains in the VDAC pore provides for the reliable quantitative prediction of the efficiency of the proposed means of MOM regulation.

## Figures and Tables

**Table 1:** Carboxy-terminal tail (CTT) sequences of human and yeast α- and β-tubulin isotypes, starting from the end of helix 12 at residue 430 [41], and α-synuclein. The negatively charged residues are highlighted in red. The total negative charge in the CTT includes the carboxyl group from the final amino acid. Adapted with permission from Rostovtseva et al., *J. Biol. Chem*. (2018). Copyright © 2018, Elsevier.

Human Tubulin CTT	Carboxy-Terminal Sequence	Number of Amino Acids	Total Negative Charge
α1	EVGVDSV EGEGEEEGEEY	18	−10
β2	QYQDATA DEQGEFEEEEGEDEA	22	−12
β3	QYQDATA EEEGEMYEDDEEESEAQGPK	27	−12
α-synuclein CTT	KKDQL GKNEEGAPQE GILEDMPVDP DNEAYEMPSE EGYQDYEPEA	45	−13

## Data Availability

The data supporting reported results can be obtained from Rostovtseva upon reasonable request.
